# The Antitoxin Protein of a Toxin-Antitoxin System from *Xylella fastidiosa* Is Secreted via Outer Membrane Vesicles

**DOI:** 10.3389/fmicb.2016.02030

**Published:** 2016-12-20

**Authors:** André da Silva Santiago, Juliano S. Mendes, Clelton A. dos Santos, Marcelo A. S. de Toledo, Lilian L. Beloti, Aline Crucello, Maria A. C. Horta, Marianna T. de Pinho Favaro, Duber M. M. Munar, Alessandra A. de Souza, Mônica A. Cotta, Anete P. de Souza

**Affiliations:** ^1^Centro de Biologia Molecular e Engenharia Genética, Instituto de Biologia, Universidade Estadual de CampinasCampinas, Brazil; ^2^Departamento de Física Aplicada, Instituto de Física Gleb Wataghin, Universidade Estadual de CampinasCampinas, Brazil; ^3^Instituto Agronômico de Campinas, IAC-APTA CitrusCordeirópolis, Brazil; ^4^Departamento de Biologia Vegetal, Instituto de Biologia, Universidade Estadual de CampinasCampinas, Brazil

**Keywords:** *Xylella fastidiosa*, protein characterization, toxin-antitoxin system, OMV, biofilm

## Abstract

The *Xylella fastidiosa* subsp *pauca* strain 9a5c is a Gram-negative, xylem-limited bacterium that is able to form a biofilm and affects citrus crops in Brazil. Some genes are considered to be involved in biofilm formation, but the specific mechanisms involved in this process remain unknown. This limited understanding of how some bacteria form biofilms is a major barrier to our comprehension of the progression of diseases caused by biofilm-producing bacteria. Several investigations have shown that the toxin-antitoxin (TA) operon is related to biofilm formation. This operon is composed of a toxin with RNAse activity and its cognate antitoxin. Previous reports have indicated that the antitoxin is able to inhibit toxin activity and modulate the expression of the operon as well as other target genes involved in oxidative stress and mobility. In this study, we characterize a toxin-antitoxin system consisting of XfMqsR and XfYgiT, respectively, from *X. fastidiosa* subsp. *pauca* strain 9a5c. These proteins display a high similarity to their homologs in *X. fastidiosa* strain Temecula and a predicted tridimensional structure that is similar to MqsR-YgiT from *Escherichia coli*. The characterization was performed using *in vitro* assays such as analytical ultracentrifugation (AUC), size exclusion chromatography, isothermal titration calorimetry, and Western blotting. Using a fluorometric assay to detect RNAses, we demonstrated that XfMqsR is thermostable and can degrade RNA. XfMqsR is inhibited by XfYgiT, which interacts with its own promoter. XfYgiT is known to be localized in the intracellular compartment; however, we provide strong evidence that *X. fastidiosa* secretes wild-type XfYgiT into the extracellular environment via outer membrane vesicles, as confirmed by Western blotting and specific immunofluorescence labeling visualized by fluorescence microscopy. Taken together, our results characterize the TA system from *X. fastidiosa* strain 9a5c, and we also discuss the possible influence of wild-type XfYgiT in the cell.

## Introduction

*Xylella fastidiosa* subsp *pauca* strain 9a5c is a Gram-negative bacteria and the causal agent of citrus variegated chlorosis (CVC). *X. fastidiosa* strain 9a5c is able to form a biofilm inside the xylem vessels of susceptible hosts, leading to xylem occlusion, nutritional deficiency, and death during the latter stages of disease. This disease leads to great economic losses of citrus crops and orange juice production in São Paulo, Brazil (Rodrigues et al., 2013). The growth of *X. fastidiosa* strain 9a5c is based on changes in the organization of cells, extracellular polymeric substance (EPS) secretion, and biofilm formation. The stages of biofilm formation by *X. fastidiosa* cells are known: days 3 and 5 correspond to the initial adhesion of the cells to a surface; microcolony formation occurs on day 10; the biofilm reaches maturation on day 20; and planktonic cells are released to initiate the cycle on day 30 (Caserta et al., 2010).

A biofilm is an association of cells surrounded by an EPS, and it is formed by diverse substances such as extracellular DNA and complex polysaccharides (Janissen et al., 2015). Biofilm formation results in water deficiency, limitations in nutrient transport and death during later stages of infection (Rodrigues et al., 2013). This structure is involved in the pathogenicity of several species such as *X. fastidiosa* (Caserta et al., 2010; Voegel et al., 2010; Janissen et al., 2015), *Neisseria meningitides* (Arenas et al., 2015), *Streptococcus pneumonia* (Domenech et al., 2015), *Salmonella enteric* (O'Leary et al., 2015), and *Pseudomonas syringae* (Chowdhury and Jagannadham, 2013), conferring resistance to antibiotics and other chemicals used to control bacterial populations.

The mechanisms underlying biofilm formation are incompletely understood. However, some genes are known to be involved in the process, including the toxin-antitoxin operon, which is also known as the TA system (Lee et al., 2014). Genes encoding the TA operon are widespread among bacteria and archaea (Gerdes and Maisonneuve, 2012). TA operons can be present on plasmids or chromosomes (Jensen and Gerdes, 1995). These genes are co-expressed under the regulation of the same promoter, which is negatively auto-regulated by antitoxin via its DNA-binding domain (Hayes and Kedzierska, 2014). Physiologically, the TA operon is involved in post-segregational killing, which can induce death in cells that fail to inherit a plasmid (Brzozowska and Zielenkiewicz, 2013; Park et al., 2013). The formation of persister cells is also induced; these cells confer antibiotic tolerance to bacterial populations that lack genetic mutations and the capacity to form biofilms (Gerdes and Maisonneuve, 2012; Germain et al., 2015).

TA systems are known to be related to the formation of persister cells in many species and also, to some extent, in the formation of biofilms (Muranaka et al., 2012; Lee et al., 2014). A previous study involving the *X. fastidiosa* strain Temecula, which is the causal agent of Pierce's disease in grapevine, demonstrated that TA systems do not play the same role in the cell. For example, in mutant assays using *dinJ*/*relE* and *ygiT*/*mqsR*, even the absence of *mqsR* led to an increase in biofilm formation of strain Temecula, whereas the *dinJ*/*relE* mutants responded to nutritional deprivation, which can be related to the survival of *X. fastidiosa* strain Temecula in the nutrient-poor environment of xylem (Lee et al., 2014).

The aim of this work was to characterize XfYgiT and XfMqsR from *X. fastidiosa* strain 9a5c; these proteins are classified in the *X. fastidiosa* database as a hypothetical protein and an HTH-type transcriptional regulator, respectively. Using bioinformatics tools for sequence prediction, we identified these proteins based on homology to the primary protein sequences. The recombinant proteins were overexpressed using an *Escherichia coli* host and purified by two-step chromatography. An initial structural analysis confirmed the secondary structures of the purified proteins. Subsequently, biochemical, thermodynamic, and hydrodynamic assays were performed to characterize the protein system. Using polyclonal antibodies against both proteins, we confirmed the expression of XfYgiT in outer membrane vesicles (OMVs) as an unusual secreted protein. Our findings supply novel information regarding the role of this system in bacterial pathogenesis.

## Materials and methods

### Cloning, expression, and purification

The open reading frames (*orfs*) *Xf2162* encoding the toxin XfMqsR (303 bp; NCBI n°. AAF85288.1) and *Xf2163* encoding the antitoxin XfYgiT (402 bp; NCBI n°. AAF85289.1) were amplified from the genomic DNA of *X. fastidiosa* strain 9a5c using specific primers. For XfYgiT, the forward primer 5′-CAAGGACATATGACCATGAGATGTCC-3′ and the reverse primer 5′-ACGGCTCGAGACTCTTCACTTCG-3′ were used for amplification. For XfMqsR, the forward primer 5′-ATGGCATATGGAGAAAGGCAC-3′ and the reverse primer 5′-GGACATCTCGAGGTCATAACTCC-3′ were used for amplification. The forward and reverse primers contained *Nde*I and *Xho*I restriction sites (underlined), respectively. After the amplification products were digested, XfYgiT was cloned into pET29a; the toxin XfMqsR was cloned into pET28a. The constructed plasmids were used to transform competent C43 (DE3) cells.

For the protein expression assays, the cells were grown at 37°C with shaking at 250 rpm. Protein expression was induced via the addition of lactose at a final concentration of 5.6 mmol.L^−1^. Lactose was added after the OD_600_ of each culture reached 0.6 (XfYgiT) or 1.0 (XfMqsR). The temperature was then reduced to 25°C, and the cells were grown with shaking at 250 rpm for 12 h. The cells were centrifuged, and the pellet was stored at −20°C.

The pellet was suspended in sodium phosphate buffer (25 mmol.L^−1^ sodium phosphate at pH 7.8, 150 mmol.L^−1^ NaCl, and 20 mmol.L^−1^ β-mercaptoethanol). The cells were disrupted via sonication. Eight cycles of 1 min of sonication and 5 min of rest were performed (Cole Parmer Ultrasonic Homogenizer 4710). The proteins were purified by affinity chromatography using a nickel-nitrilotriacetic acid (Ni-NTA) column. The supernatant fractions were applied to a Ni-NTA column, and the protein was eluted using an imidazole gradient. The toxin and the antitoxin were eluted at imidazole concentrations of 50 mmol.L^−1^ and 100 mmol.L^−1^, respectively. Both proteins were separated from contaminants using a Superdex 200 10/300 (GE Healthcare) gel filtration column as described below.

### Analytical size-exclusion chromatography (SEC)

Analytical size-exclusion chromatography (SEC) was used to determine the oligomeric conformations of both proteins. A Superdex 200 10/300 pre-packed column (GE Life Science) was used at a flow rate of 0.4 mL.min^−1^. After equilibration with sodium phosphate buffer, 7 μmol.L^−1^ of each protein was loaded onto a column. A UV-VIS flow cell was used for protein detection at 280 nm. To obtain the XfMqsR-XfYgiT complex, the proteins were mixed at equimolar concentrations (7 μmol.L^−1^ each) and incubated for 12 h at 25°C; the protein mixture was then applied at a 1:1 ratio to the column under the same conditions described above.

### Analytical ultracentrifugation (AUC) measurements

Sedimentation velocity experiments using XfYgiT, XfMqsR, and the XfYgiT-XfMqsR complex were performed at concentrations ranging from 0.2 to 1 mg.mL^−1^ in sodium phosphate buffer (25 mmol.L^−1^ sodium phosphate at pH 7.8, 150 mmol.L^−1^NaCl, and 20 mmol.L^−1^ β-mercaptoethanol). A Beckman Optima XL-A analytical ultracentrifuge was used. Data acquisition during analytical ultracentrifugation (AUC) was performed at 280 nm, 20°C, and 35,000 rpm using an AN-60Ti rotor. The AUC data analyses were performed using SedFit software (Version 12.1). The experimental s-value was calculated for the standard sedimentation coefficient at a protein concentration of 0 mg.mL-1 of protein (${\text{s}}_{20,\text{w}}^{0}$) to prevent interference due to the buffer density, viscosity and temperature. We used linear fitting of the curve of the ${\text{S}}_{20,\text{w}}^{0}$ value as a function of the protein concentration. The buffer viscosity (η = 1.0289 × 10^−2^ poise), buffer density (ρ = 1.008 g.mL^−1^) and partial-specific volume (Vbar = 0.730787 mL.g^−1^ for XfYgiT, 0.741942 mL.g^−1^ for XfMqsR, and 0.731885 mL.g^−1^ for the XfYgiT-XfMqsR complex) used for analysis were estimated using the Sednterp server (http://sednterp.unh.edu/). The R_s_, MMpred, and ${\text{s}}_{20,\text{w}}^{0}$ for the recombinant purified proteins were obtained from the AUC data analyses using SedFit.

### Circular dichroism (CD) and thermally-induced unfolding

The protein fractions were dialyzed in sodium phosphate buffer (10 mmol.L^−1^ sodium phosphate at pH 7.8) and 1 mmol.L^−1^ tris-2-carboxyethyl-phosphine (TCEP). The far-UV circular dichroism (CD) spectra of recombinant XfMqsR and XfYgiT were measured using a JASCO J-810 spectropolarimeter (Dichrograph; Japan Spectroscopic; Japan). The assays were performed in a quartz cuvette with a path length of 1 mm. Ten measurements were recorded at 222 nm at a rate of 50 nm.min^−1^ at 25°C. The toxin and antitoxin concentrations were 21 and 20 μmol.L^−1^, respectively.

Each protein was unfolded separately at a concentration of 10 μmol.L^−1^ to obtain the melting temperature (*Tm*). The proteins were then mixed and incubated for 12 h at 25°C to enable their interaction. Both proteins were subjected to the same experimental conditions. The temperature was adjusted from 20° to 90°C at 1°C per minute, followed by a decrease of 1°C per minute until the temperature reached 20°C.

### Isothermal titration calorimetry (ITC)

The isothermal titration calorimetry (ITC) experiments were performed at 25°C using an Auto iTC200 calorimeter (MicroCal, Northampton, MA, USA). To determine the binding constant and the heat of the interaction, the recombinant proteins were extensively dialyzed against sodium phosphate buffer (25 mmol.L^−1^sodium phosphate at pH 7.8, 150 mmol.L^−1^NaCl, and 0.3 mmol.L^−1^TCEP) at 25°C. Thirty-three injections of 1 μL of XfMqsR at 100 μmol.L^−1^ were titrated into ~204 μL of XfYgiT at 10 μmol.L^−1^, with 300 s intervals between each injection.

The obtained heat signals from the raw ITC data were integrated using Origin software (MicroCal Inc., Northampton, MA). The heat from the reference buffer and the titrant, which is the heat due to the diffusion of the protein into the buffer, was subtracted to calculate the corrected heat release. A single-site binding isotherm model was used to match the data and to assess the dissociation affinity (K_d_), the enthalpy (ΔH), the entropy (ΔS), and the binding stoichiometry (N).

### RNase activity assay

An RNAse AlertKit (Invitrogen, Carlsbad, CA, USA) was used to investigate the RNAse activity of XfMqsR. The reactions were performed in 96-well-flat-bottom plates in a total volume of 50 μL. A total of 5 μmol.L^−1^ of each recombinant protein was mixed with 5 μL of fluorescent substrate and 5 μL of 10X RNase Alert lab test buffer. After addition of the protein, the plate was immediately placed in the fluorometer (2300 EnSpire Multimode Plate Reader, Perkin-Elmer). The reactions were monitored for 200 min using excitation/emission wavelengths of 490/520 nm. The proteins were solubilized in buffer containing 25 mmol.L^−1^ phosphate at pH 7.8 and 150 mmol.L^−1^ NaCl. RNAse A was used as a positive control according to the manufacturer's instructions.

The RNAse activity was further visualized on an agarose gel. Increasing concentrations of XfMqsR were mixed with 800 ng of total RNA from *X. fastidiosa* and incubated for 30 min at 25°C. The RNA was then applied to a 1% denaturing agarose gel at 80 V for 2 h.

### Growth conditions and protein extraction

*X. fastidiosa* cells were inoculated into 50 mL of periwinkle wilt GelRite or PWG broth (0.4% w.v^−1^ phytone peptone, 0.1% w.v^−1^ trypticase peptone, 7.35 mmol.L^−1^KH_2_PO_4_, 6.89 mmol.L^−1^ K_2_HPO_4_, 1.62 mmol.L^−1^, MgSO_4_, 0.001% w.v^−1^ hemin chloride, 0.002% g.L^−1^phenol red, 0.4% w.v^−1^ glutamine, 0.6% w.v^−1^ BSA pH 6.8) at an initial OD_600_ of 0.3. The cultures were incubated at 150 rpm and 25°C. The cells were collected at 10, 20, or 30 days after inoculation, and total cellular proteins were extracted via sonication in buffer (50 mmol.L^−1^ sodium phosphate, 300 mmol.L^−1^, NaCl, 0.2%, Tween-20, 20 mmol.L^−1^β-mercaptoethanol, and 3 mmol.L^−1^ EDTA). An Ultrasonic Homogenizer 4710 Series (Cole Parmer) set to 70% was used for four rounds of sonication of 20 s each.

### Detection of XfYgiT and XfMqsR by western blotting

The extracts were quantified using a Pierce BCA Protein Assay Kit (Rockford, IL, USA) according to the manufacturer's instructions. Next, 100 μg of total protein was applied to a 1% SDS-PAGE gel. After migration, the proteins were transferred to a nitrocellulose membrane using a Trans-Blot SD Semi-Dry apparatus (Bio-Rad) with a constant voltage of 25 V for 20 min. The membrane was blocked with 5% BSA for 8 h in TTBS buffer (0.1% Tween-20, 136 mmol.L^−1^ NaCl, 2.6 mmol.L^−1^ KCl, and 25 mmol.L^−1^Tris, pH 7.5). The solution was replaced with a primary antibody obtained from the sera of rabbits immunized with the heterologous protein (diluted 1:4000 for XfMqsR, 1:1000 for XfYgiT, and 1:8000 for XfPal); the membrane was then incubated overnight. The secondary antibody (an alkaline phosphatase-conjugated anti-rabbit IgG diluted 1:5000) was applied for 3 h. Subsequently, the membrane was incubated in revelation buffer for 20 min, 50 μL NBT/BCIP solution (Sigma-Aldrich) were added and the membrane was monitored until bands were visible. Primary antibodies were generated by Rheabiotech® (Campinas, Brazil) in rabbits using heterologous proteins as antigens until blood serum extraction.

A polyclonal antibody against XfPal, a well-known peptidoglycan-associated lipoprotein present in OMVs, served as a positive control in the Western blotting assays. PAL is a peptidoglycan-associated outer membrane-anchored lipoprotein that is involved in cell membrane integrity and OMV formation in *Escherichia coli* (Kaparakis-Liaskos and Ferrero, 2015; Turner et al., 2015).

### Detection of XfYgiT in the secretome

*X. fastidiosa* was used to inoculate 50 mL of PWG broth. The cells were collected by centrifugation at 4000 rpm after 10, 20, and 30 days of incubation. The supernatants were then filtered using 0.22-μm membranes to completely remove the bacteria. The supernatant was lyophilized and suspended in 4 mL of water. An aliquot of 1 mL was treated with 100 mmol.L^−1^ dithiothreitol for 1 h and 300 mmol.L^−1^ iodoacetamide for 30 min. Then, 75 ng of trypsin was added for digestion *in solution*.

The peptides were chromatographically separated in a nanoAcquity UPLC (WATERS) over two columns in tandem with a Q-TOF-Micro mass spectrometer (Micromass). The first column (referred to as the trapping column) was 5 × 180 × 20 mm. The second column was 1.7 × 100 μm × 100 mm. The samples were eluted at a flow rate of 0.6 μL/min for 50 min. An acetonitrile gradient was set up as follows: an initial elution with 1% v/v acetonitrile for 1 min, an acetonitrile gradient increasing from 1 to 50% up to 40 min and increasing from 50 to 85% up to 45 min, 85% acetonitrile for 2 min, and then an acetonitrile gradient decreasing to 1% until completion at 50 min.

The peptides were ionized at 3000 V and fragmented using collision energy ranging from 20 to 95 eV according to the *m/z* and the size of the peptides. A charge state ranging from 2+ to 4+ was considered.

The spectra containing the fragmentation patterns were analyzed using ProteinLynx Global Server 2.4 software (WATERS). The peptide sequences were compared using the SwissProt database for tryptic digestion products. A missed cleavage at up to one site and a maximal error of 30 ppm were considered.

### Purification and visualization of OMVs

OMV separation was accomplished based on a previously described method (Voegel et al., 2010). The bacteria were centrifuged, and the supernatant was recovered and filtered using a 0.22-μm membrane. Filtration was performed to remove any cells that remained in suspension; *X. fastidiosa* is a rod-shaped bacterium with a radius of 0.25 to 0.35 μm and a length of 0.9 to 3.5 μm (Wells et al., 1987). The resulting solution was lyophilized for 2 days, and the dry powder was suspended in 4 mL PBS (137 mmol.L^−1^ NaCl, 2.7 mmol.L^−1^ KCl, 10 mmol.L^−1^ Na_2_HPO_4_, 2 mmol.L^−1^ KH_2_PO_4_) and centrifuged twice at 16,000 × g for 20 min to remove the cell debris. The final supernatant was centrifuged at 100,000 × g for 4 h, the supernatant was transferred to a new tube and the pellet was washed with PBS and centrifuged one more time at 100,000 × g for 4 h using an L8-80M Ultracentrifuge (Beckman Coulter®, California, USA). The pellet was then suspended in 200 μL of PBS.

A 40-μL aliquot was reserved for visualization via transmission electron microscopy. Copper grids with a 200 mesh and a formvar-carbon coating (Ted Pella®, Redding, CA) were used to visualize OMVs as described in Nevot et al. (2006). First, the samples were adsorbed onto the grids by immersing the grids in 20 μL of the sample for 5 min. Then, the grids were washed via flotation in 20 μL of deionized water for 2 min. The samples were negatively stained by floating the grids on a drop of 2% (w/v) uranyl acetate for 5 min. After the grids were dried for 24 h, the OMVs were visualized under a LEO 906 transmission electron microscope at a magnification of 60 kV and 167,000 times.

### OMV protein extraction and western blotting

The pellet providing from centrifugation at 100,000 g was treated with lysozyme for 10 min and then suspended in 150 μL of Laemmli buffer (0.1% β-mercaptoethanol, 0.0005% Bromophenol blue, 10% glycerol, 2% SDS, and 63 mmol.L^−1^ Tris-HCl, pH 6.8). The samples were sonicated three times for 5 s each using a Cole Parmer 4710 series ultrasonic homogenizer. One hundred micrograms of the extract was subjected to SDS-PAGE and Western blotting as described above using polyclonal antibodies against XfYgiT, XfMqsR, and XfPal.

### *Xylella fastidiosa* 9a5c fixation and immunofluorescence labeling

For the immunofluorescence analysis using polyclonal anti-XfYgiT antibodies coupled to FITC (Rheabiotech, Campinas, Brazil), *X. fastidiosa* 9a5c bacteria were chemically fixed onto borosilicate glass based on a modified method described by Louise Meyer et al. (2010) and Bearinger et al. (2009). To covalently tether the bacteria, each glass was amino-functionalized using 0.5 mL 5 mol.L^−1^ ethanolamine in dimethylsulfoxide (DMSO) overnight at room temperature (RT). After washing three times with deionized water, 600 μL of a pre-inoculum of *X. fastidiosa* strain 9a5c grown for 10 days (O.D. 0.6) was transferred to the amino-functionalized glass. 2.4 mL fresh PW broth was added and the glass was incubated for 4 days until labeling. Subsequently, the solution was removed and 2 mL of MES (pH 4.8) containing 50 mM EDC [1-ethyl-3-(3-dimethylaminopropyl)carbodiimide] was added for 1 h at room temperature. After incubation, the glasses were washed three times with PBS buffer (pH 7.4) for 5 min each.

To permeabilize the peptidoglycan layer of the Gram-negative bacteria and the OMVs for adequate immunolabeling, 500 μl of PBS buffer containing lysozyme (5 mg.mL^−1^) was added to each well and the samples were incubated for 5 min. Subsequently, the wells were washed three times with PBS for 5 min each. To reduce non-specific adsorption of the specific anti-XfYgiT antibodies, 500 μl of PBS-BSA (2%) blocking solution was added to the wells and the samples were incubated for 1 h at RT. After washing the wells three times with PBS-Tween 20 (0.025%) for 5 min each, polyclonal anti-XfYgiT antibodies coupled to FITC were added to the wells at a concentration of 1:400 in PBS-BSA (2%) for 1 h at 37°C. In a final step, the wells were washed three times for 5 min each with PBS, once with PBS-Tween 20 (0.025) and then twice with deionized water. The glasses were then dried at room temperature before visualization.

For positive control, antibodies against XfPal was labeled with Atto 594 and for negative control, anti-XfMqsR was labeled with Atto 488. The samples were treated the same way as explained to XfYgiT. Due to fluorophore compatibility, simultaneous labeling were performed using anti-XfYgiT and XfPal; XfYgiT and XfMqsR.

### Immunofluorescence measurements

To localize the target protein via immunofluorescence labeling, the samples were measured using an epi-fluorescence microscope (Nikon TE2000U, USA) with a Peltier cooled back-illuminated EMCCD camera (Andor IXON^3^, 1024 × 1024 pixels, Ireland) for sensitive fluorescence detection. FITC-fluorophor excitation was achieved using a 150 W Mercury lamp with filter sets (AHF, Tübingen, Germany) for blue light excitation (488 nm) and neutral density (ND) filters for bright-field observation. For each immunolabeled sample, the bacterial shape was measured using fluorescence measurements in the green wavelength range (525 nm) to localize the FITC conjugated-primary antibody. The obtained fluorescence images were further analyzed by fluorescence intensity surface plots in which the intensity thresholds were adjusted for appropriate protein location within bacteria and, additionally, to visualize the shape of *X. fastidiosa* strain 9a5c based on the auto-fluorescence emission.

### Scanning electron microscopy (SEM) preparation and visualization

Cells grown in PWG broth and the pellet after 100,000 g centrifugation were directly transferred to silica slides and incubated for 16 h to promote more adsorption. Afterwards, the slides were dried using critical point dryer CPD030 (Balzers) and deposited onto the surface of SEM pin stubs. The slides were subsequently sputtered-coated with iridium. Finally, the samples were analyzed in a Quanta FEG 250 (FEI company) field emission scanning electron microscope operated at 10 kV.

### Atomic force microscopy (AFM)

The fraction proceeded from 100,000 g centrifugation was spread in glass slides and dried under gentle nitrogen flow. In the next day, the slides were imaged with a NX-10 (Park Systems) in a low (10%) humidity chamber. AFM images were acquired using intermittent contact mode, at 0.5 Hz, with NCHR (Nanosensors) cantilevers (spring constant 42 N/m and resonance frequency in air 320 kHz).

### Ethics statement

We confirm that no specific permits were required for the described field studies. The collections were performed at two research institutions (University of Campinas and the Citriculture Center in Cordeirópolis, São Paulo, Brazil.). The bacterial cultures were grown in a certified biosafety level 1 laboratory. We confirm that this manuscript is a result of a basic research project that was developed primarily at the university and mostly funded by public funding agencies with the aim of generating new knowledge; the results should be shared with the scientific and technological community through openly available university theses and manuscripts in conventional scientific journals. We confirm that this study did not involve endangered or protected species.

## Results

### Identification of XfMqsR/XfYgiT proteins based on sequence similarity

The XfMqsR and XfYgiT sequences are annotated as a conserved hypothetical protein and an HTH-type transcription regulator, respectively, in the *X. fastidiosa* strain 9a5c database at http://www.xylella.lncc.br/. The XfYgiT presents 99% similarity to its cognate sequence in *X. fastidiosa* strain Temecula, with the replacement of isoleucine by a valine amino acid residue at position 77. XfMqsR also presents 99% similarity, with only one difference at position 6, in which strain 9a5c possesses a proline and strain Temecula has a serine (data not shown).

**Figure 1:**
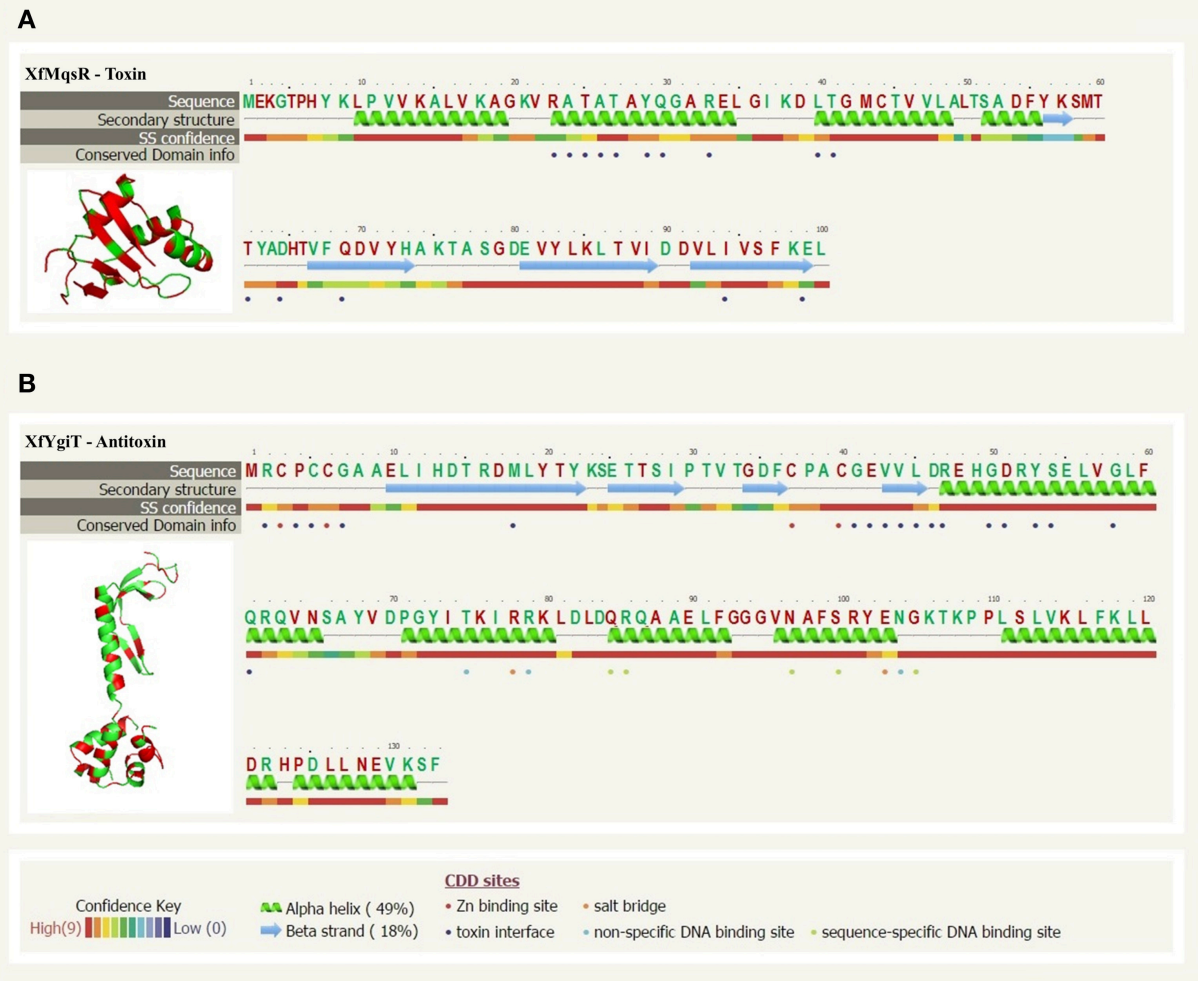
**Sequence alignment analyses and protein structure prediction of XfMqsR and XfYgiT. (A)** The structural prediction based on amino acid sequences of XfMqsR (NCBI Reference Sequence AAF85288.1) and the *E. coli* protein MqsR (PDB ID 3HI2) obtained using the PhyreV2 server with 100% confidence. This alignment revealed significant homology (60%) between the two proteins. Identical amino acid residues are shown in red. The residues corresponding to *E. coli* protein residues involved in the protein-protein interaction are indicated by violet circles. **(B)** The prediction based on the amino acid sequences of XfYgiT (NCBI Reference Sequence AAF85289.1) and the *E. coli* protein MqsA/YgiT (PDB ID C3GN) was obtained using the PhyreV2 server. Significant homology (42%) was observed. Identical amino acid residues are indicated in red. The two zinc molecules are indicated by red circles. The residues predicted to be involved in the interaction with XfMqsR are marked with violet circles; these residues correspond to *E. coli* protein residues involved in the protein-protein interaction. The residues involved in promoter recognition, which corresponded to residues in the *E. coli* protein, and the HTH are indicated by green circles.

A comparative analysis using the annotated protein sequences available in the public database and alignment tools allowed us to predict the likely tridimensional structures of the proteins based on the most similar proteins with solved structures in the protein database. The XfMqsR and XfYgiT protein sequences shared 61% sequence similarity (Figure 1A) and 42% sequence similarity (Figure 1B), respectively, with their homologs from *E. coli*, MqsR, and MqsA, respectively. A structural modeling prediction using PhyreV5 2.0 software (http://www.sbg.bio.ic.ac.uk/phyre2/html/page.cgi?id=index) (Kelley and Sternberg, 2009) revealed a high similarity with the respective MqsR and MqsA from *E.coli* (Figure 1) with 100% confidence (the probability that the primary sequences of XfMqsR and XfYgiT are homologous) (Figure 1).

Sequence alignment analysis using the primary protein sequences and models of the MqsR/MqsA system from *E. coli* revealed that the XfYgiT protein possesses two domains: a putative CXXC zinc-finger domain at the N-terminus (residues 3–6 and 37–40) and a well-conserved DNA-binding domain containing a XRE-HTH motif at the C-terminus encompassing helices α2, α3, α4, and α5 (residues 74–127). These N- and C-termini domains displayed 28% and 59% similarity, respectively, with the homologous *E. coli* protein (Figure 1B). The most highly conserved residues in XfMqsR were located in the β-sheet regions. Although RNAse activity was detected, the primary sequence did not display similarity to the general RNAse domain contained by, for instance, RNAse A and RNAse H. However, we observed structural similarity to the RNAses of other toxin-antitoxin operons, YoeB and RelE; these proteins are toxins of the TA system from *E. coli* (Brown et al., 2009). No domains in the toxin were predicted to be involved in RNAse activity or protein-protein interactions. However, some of the amino acid residues involved in RNAse activity in *E.coli* are conserved in *X. fastidiosa* strain 9a5c; these include Lys^56^, Gln^68^, Tyr^81^, and Lys^96^ (Brown et al., 2009).

### Cloning, expression, and purification

**Figure 2:**
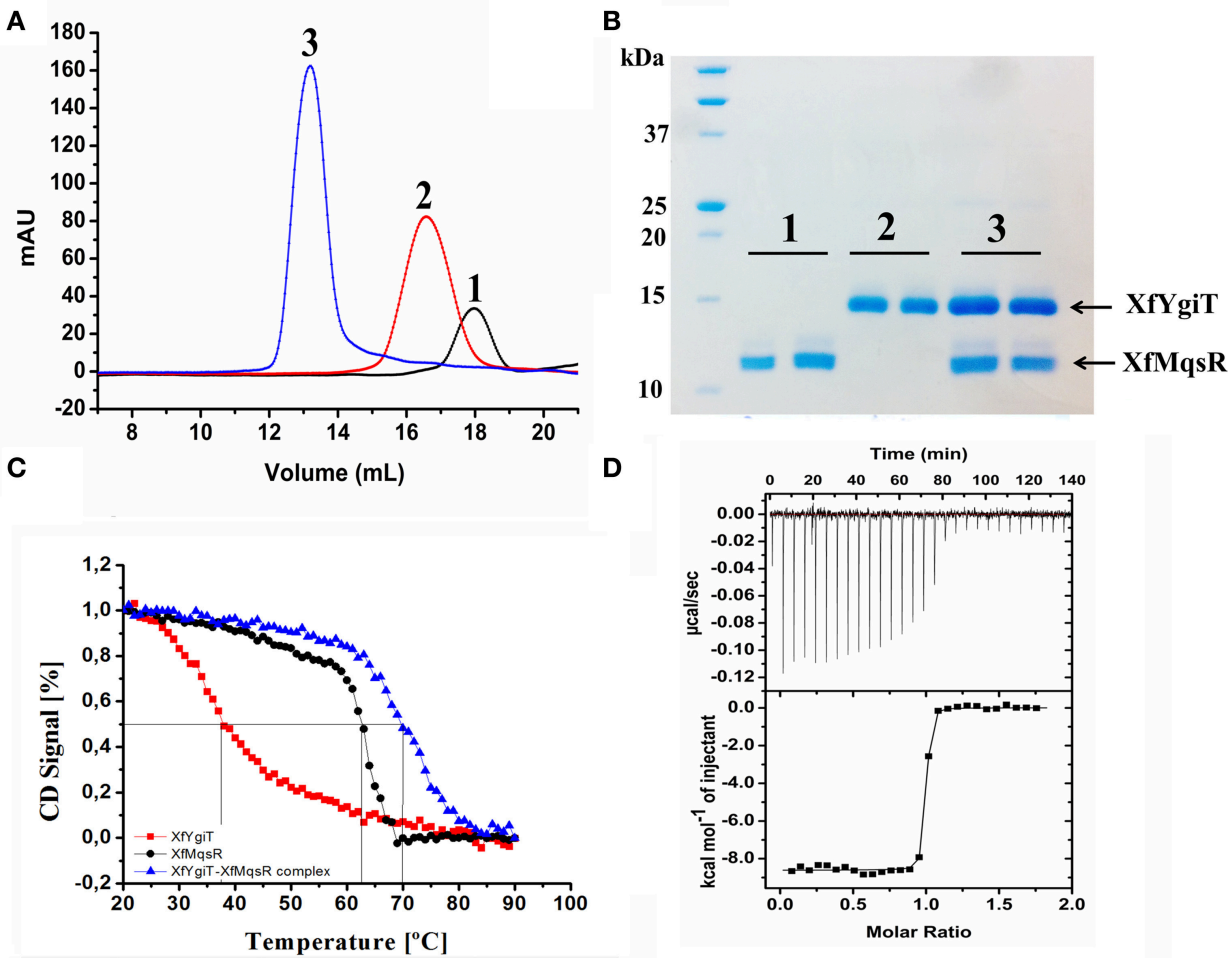
**Hydrodynamic properties of the interaction between XfMqsR and XfYgiT. (A)** Chromatograms of the analytical size-exclusion chromatography experiments for the recombinant purified toxin and antitoxin. The toxin and antitoxin are represented by the black and red lines, respectively. The complex is represented by the blue line. **(B)** SDS-PAGE (15%) of the fractions eluted during the analytical size-exclusion chromatography experiment. The molecular weight of XfMqsR was 13.1 kDa; the molecular weight of XfYgiT was 15.94 kDa. **(C)** Analysis of complex thermostability. The spectra at 222 nm reflect a loss of protein structure with increasing temperature. The *Tm*s obtained were 37°C for XfYgiT (black line), 62.5°C for the toxin (red line), and 70°C for the complex (blue line). **(D)** Determination of the dissociation constant (Kd) of the interaction based on ITC. The characteristics of the interaction curve revealed a high-affinity, thermodynamically favorable interaction at a protein molar ratio of 1:1; for this interaction, ΔH was −8609 ± 36.51cal/mol and ΔS was 11.8 cal/mol/deg.

Positive colonies containing the respective inserts were selected, and the induced recombinant proteins were prepared with high purity using size-exclusion chromatography (Figure 2A). After the *orf* sequences were analyzed, XfMqsR was cloned into pET28a and XfYgiT was cloned into pET29a. The former clone produced a 13.12-kDa His-tagged protein with a theoretic pI of 8.7.The latter clone produced a 15.94 kDa His-tagged protein with a theoretic pI of 6.74. The insertion of the His-tag at the C-terminal end of XfYgiT in pET29a was critical for the appropriate conformation of the N-terminus. When the protein was cloned into pET28a, which introduces a His-tag at the N-terminus, the conformation of the region containing the zinc-finger was disrupted, resulting in protein aggregation and precipitation, as observed by SEC (data not shown).

Both proteins were purified using the same two methods (Ni-NTA affinity chromatography and SEC) to remove undesirable proteins. These purification processes resulted in protein solutions of high purity. Purification of XfYgiT and XfMqsR resulted in yields of 10 and 0.4 mg.L^−1^, respectively.

### Hydrodynamic characterization of the recombinant proteins using SEC and AUC

To confirm the formation of a stable complex *in vitro*, the purified recombinant proteins were mixed at a 1:1 ratio (7 μmol.L^−1^ each). This mixture was loaded onto a Superdex 200 10/300 GL column and analyzed by SEC. Detection of a fraction representing the complex demonstrated that the proteins were completely bound at a 1:1 ratio following a 12-h incubation; no peaks representing the isolated proteins were observed (Figure 2A). The peaks corresponding to the XfYgiT-XfMqsR complex were eluted at retention volumes of 13.5, 16, and 18 mL. When fitted to the calibration curve generated using the protein standards, the obtained apparent molecular masses (MMapp) were 31.6 kDa for XfYgiT and 16.9 kDa for XfMqsR; these weights approximately corresponded to dimeric and monomeric forms, respectively (data not shown). It is worth noting that the intrinsic properties of analytical SEC can interfere with MMapp estimations; such properties include buffer constitution, protein shape, and hydrophobic interactions between proteins and the column resin. This interference can lead to errors during the calculation of the protein molecular mass. Thus, an additional measurement was obtained by AUC to confirm the MMapp. The proteins eluted at the peaks were separated by 15% SDS-PAGE (Figure 2B), and the results confirmed that both XfYgiT and XfMqsR were present in the complex.

**Figure 3:**
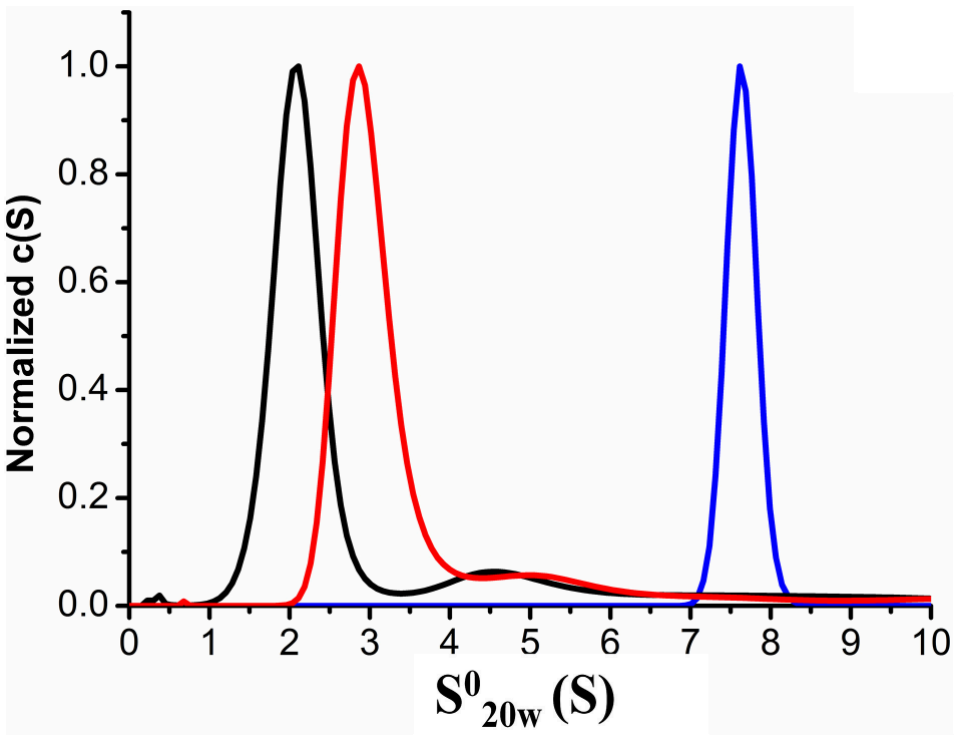
**Analytical ultracentrifugation properties of XfYcjZ-like proteins**. Sedimentation velocity AUC experiments using heterologous proteins at a range of concentrations from 0.2 to 1 mg.mL^−1^. The sedimentation profiles show that one species predominated; this species corresponded to the XfMqsR monomer (black line), which had a ${\text{s}}_{20,\text{w}}^{0}$ (S) value of 2.073. Dimeric XfYgiT (red line) presented a ${\text{s}}_{20,\text{w}}^{0}$ (S) value of 2.939. The dimer of the XfYgiT-XfMqsR complex (blue line) presented a ${\text{s}}_{20,\text{w}}^{0}$ (S) value of 7.602.

We also employed AUC to confirm the characteristics of the purified recombinant proteins XfMqsR and XfYgiT and the XfYgiT-XfMqsR complex in solution. An analysis of the AUC data indicated that XfYgiT, XfMqsR and the complex sedimented as single species with ${\text{s}}_{20,\text{w}}^{0}$ values of 2.939 ± 0.1, 2.073 ± 0.08, and 7.602 ± 0.15 (Figure 3), respectively. The experimental molecular masses (MM_exp_) for XfYgiT, XfMqsR and the complex were 32.5 ± 0.2, 10.1 ± 0.6, and 58.36 ± 1.4 kDa, respectively. These results suggest that in solution, XfYgiT behaves as a dimer, XfMqsR behaves as a monomer, and the XfYgiT-XfMqsR complex interacts at a 2:2 ratio. The AUC data corroborated the analytical SEC results, suggesting that XfYgiT and XfMqsR interact at an equimolar ratio.

Regarding the frictional ratio (*f/f*_0_), which is related to the protein shape, XfYgiT presented an *f/f*_0_ equivalent to 1.6, which suggests that XfYgiT is a moderately elongated protein. XfMqsR and the complex presented with *f/f*_0_ ratios equivalent to 1.14 and 1.41, respectively; these values suggest that the molecules possess globular shapes (Erickson, 2009). The oligomeric states of the homologous *E. coli* proteins in solution have been described; MqsA forms a dimer, and MqsR persists as a monomer. The complex is formed by a dimer of MqsA and two monomers of MqsR, as observed in a crystallography assay (Brown et al., 2009).

### Thermal stability analysis based on CD

Both proteins absorbed polarized light at 222 nm; this wavelength was used to analyze unfolding due to increasing temperature. XfYgiT showed a *Tm* of 37.8°C and displayed a high capacity to recover its secondary structure. Approximately 86% of the protein that unfolded at 90°C was able to regain its α-helix structure when the temperature returned to 20°C. XfMqsR displayed a high *Tm* (62.5°C). However, this protein was unable to recover any of its secondary structure after unfolding at 90°C. Surprisingly, the complex exhibited a high level of thermostability and a higher *Tm* (70.5°C) than either protein alone (Figure 2C).

### ITC reveals a high affinity interaction between XfMqsR and XfYgiT

After subtracting the heat provided by the buffer, we obtained the specific heat of the interaction at the molar ratio involved in complex formation. The interaction was characterized by strong binding due to a pronounced slope, revealing nanomolar affinity. After fitting, the ITC experiment revealed a dissociation constant of 0.785 ± 0.241 nmol.L^−1^and a stoichiometry of 1:1. We found that ΔH was −8609 ± 36.51 and that ΔS was 11.8 cal/mol/deg, indicating that complex formation is thermodynamically favorable (Figure 2D).

### Fluorimetric RNAse activity assay

**Figure 4:**
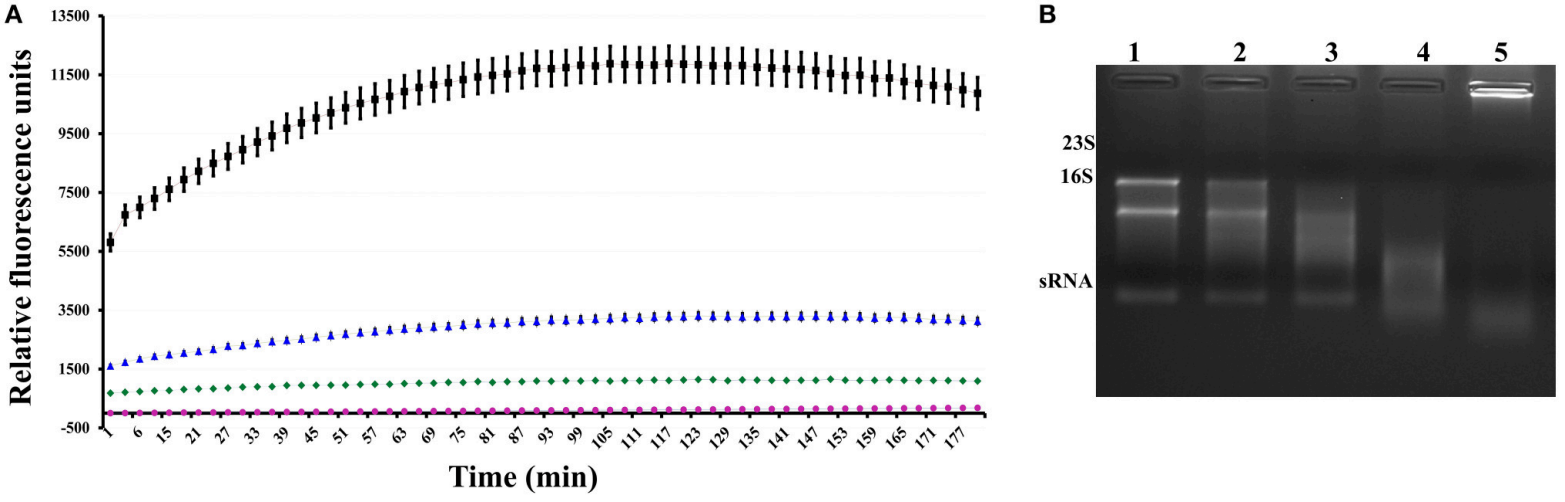
**Assessment of the RNAse activity of the toxin. (A)** Fluorimetric measurement of the activity of the toxin and its inhibition by the antitoxin. The red line represents the negative control, and the pink line represents toxin inhibition by the antitoxin. The blue line represents toxin activity, and the black line represents the activity of the positive control, RNAse A. **(B)** Visualization of digested RNA in a 1% denaturing agarose gel in the presence of increasing concentrations of XfMqsR. Lane 1, 800 ng of total RNA without XfMqsR. Lane 2, 800 ng of total RNA with 1 μmol.L^−1^ XfMqsR. Lane 3, 800 ng of total RNA with 2 μmol.L^−1^ XfMqsR. Lane 4, 800 ng of total RNA with 5 μmol.L^−1^ XfMqsR; Lane 5, 800 ng of total RNA with 10 μmol.L^−1^ XfMqsR.

After we confirmed the interaction between these proteins, we performed a quantitative analysis to measure RNAse activity. To achieve this goal, the purified heterologous XfMqsR protein was assessed using an RNAseAlert Kit. XfMqsR showed RNAse activity; however, its activity was less than that of the positive control, RNAseA. XfMqsR is sequence-specific and shows a preference for cleavage at sites G_▴_CU and, to a minor degree, at G_▴_CC (Lee et al., 2014), whereas RNAse A cleaves the phosphodiester bond between the 5′-ribose of a nucleotide and the phosphate group attached to the 3′-ribose of an adjacent pyrimidine nucleotide. XfYgiT was able to inhibit XfMqsR activity; the relative fluorescence observed when both proteins were present was lower than that emitted in the presence of XfMqsR alone. There was no RNAse contamination because no fluorescence was released from the negative control (Figure 4A). RNAse activity was also visualized in an agarose gel, in which the bands corresponding to the 23S, 16S and small RNAs (sRNAs) were completely digested by XfMqsR (Figure 4B).

### Detection of XfYgiT via MS/MS and western blot analysis

**Figure 5:**
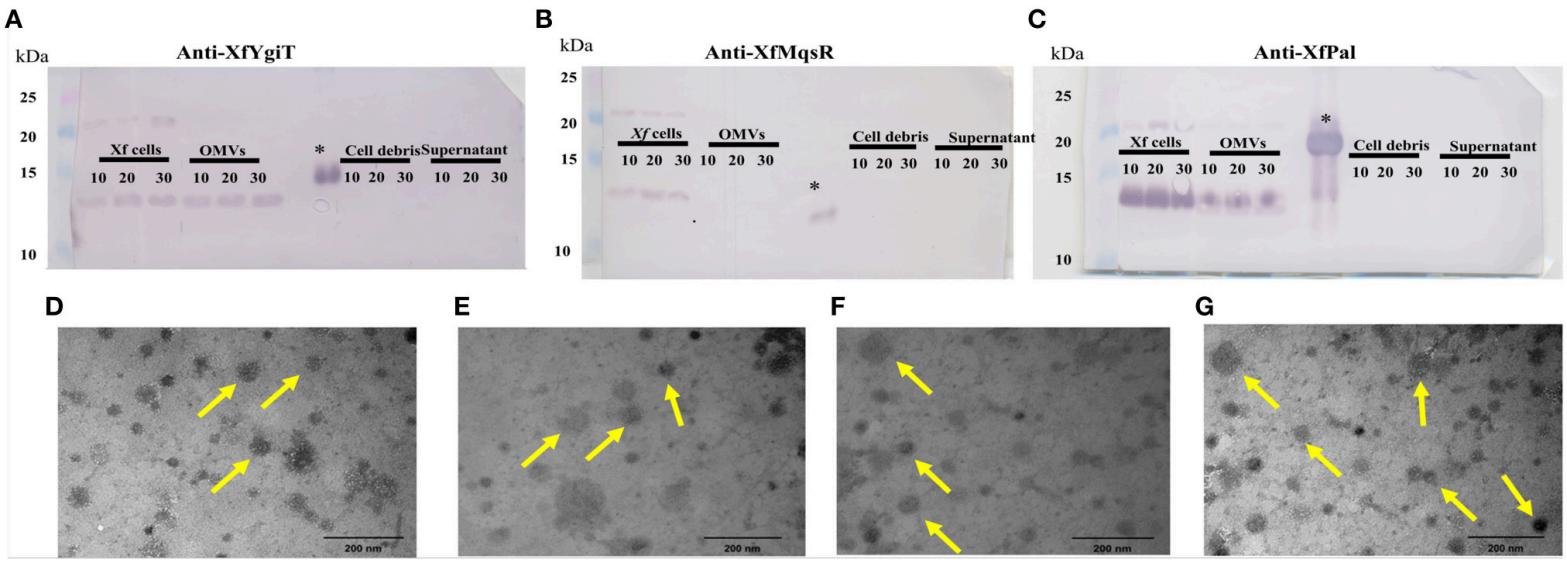
**Immunodetection of XfMqsR, XfYgiT, and XfPal in ***X. fastidiosa*** in the fractions corresponding to the total cell proteins, OMVs, cell debris, and supernatant after OMV centrifugation**. Total proteins were quantified using the BCA method, and 100 μg of protein were separated in a 15% SDS-PAGE gel. Western blots were performed using polyclonal antibodies against XfYgiT **(A)**, XfMqsR **(B)**, and XfPal **(C)**. Numbers below the lines indicate the day on which the samples were collected. Bands at line 1 correspond to the *X. fastidiosa* proteins present on days 10, 20, and 30, respectively. Bands at line 2 correspond to the proteins in the OMV pellet. Bands at line 3 correspond to the supernatant collected after OMV centrifugation, and bands at line 4 correspond to the cell debris fraction. XfPal was chosen as a positive control because this protein is involved in OMV formation. The figures **(D–G)** represent visualization, using LEO 906 transmission electron microscope, of the OMVs in the final step of the centrifugation at 100.000 × g.

To evaluate the possibility that XfYgiT and XfMqsR are secreted into the extracellular media, we investigated the extracellular proteins using MS/MS. The presence of four peptides that matched *orf2163* (Table 1), the XfYgiT-coding *orf* in the *X. fastidiosa* database, was detected among the secreted proteins. After isolating the OMVs via ultracentrifugation, the homogeneity of the preparation was evaluated using transmission electron microscopy (Figures 5D–G). After confirming the absence of *X. fastidiosa* cells, the fractions corresponding to the extracellular proteins and the OMVs were subjected to SDS-PAGE followed by Western blotting. We confirmed the presence of XfYgiT in the OMVs. Bands corresponding to wild-type XfYgiT (15 kDa) were identified by Western blotting on days 10, 20, and 30 and in the OMV fraction (Figure 5A). However, XfYgiT was not detected in the extracellular media (Figure 5B). Moreover, XfMqsR was detected in the total cellular protein, while no bands corresponding to XfMqsR were detected in either the OMVs or the extracellular media (Figure 5B). The wild-type protein was detected at 14 kDa, though the expected size was 11 kDa; the heterologous XfMqsR was 13.2 kDa, as expected. As documented in previous works, peptidoglycan-associated lipoprotein is involved in the formation of OMVs (Santos et al., 2015; Turner et al., 2015). An antibody against the heterologous XfPal was used to confirm its presence in the fractions during the isolation of OMVs (Santos et al., 2015). Wild-type XfPal with a size of 14 kDa was also detected in the total cellular protein and in the OMV fractions (Figure 5C).

**Table 1:** **Peptides identified by MS/MS corresponding to the ***X. fastidiosa*** XfYgit**.

**Peptide**	***m/z***	**Molecular mass (Da)**	**Charge**
CPCCGAAELIHDTR	553.9035	1658.6963	+2
YSELVGLFQR	606.3224	1210.6346	+2
QVNSAYVDPGYITK	777.8915	1553.7726	+2
KLDLDQR	444.2489	886.4872	+2
MRCPCCGAAELIHDTRDMLYTYKSETTSIPTVTGDFCPA CGEVVLDREHGDRYSELVGLFQRQVNSAYVDPGYITKIRRKLDL DQRQAAELFGGGVNAFSRYENGKTKPPLSLVKLFKLLDRHPDLLNEVKSF			

### Fluorescence microscopy assay

**Figure 6:**
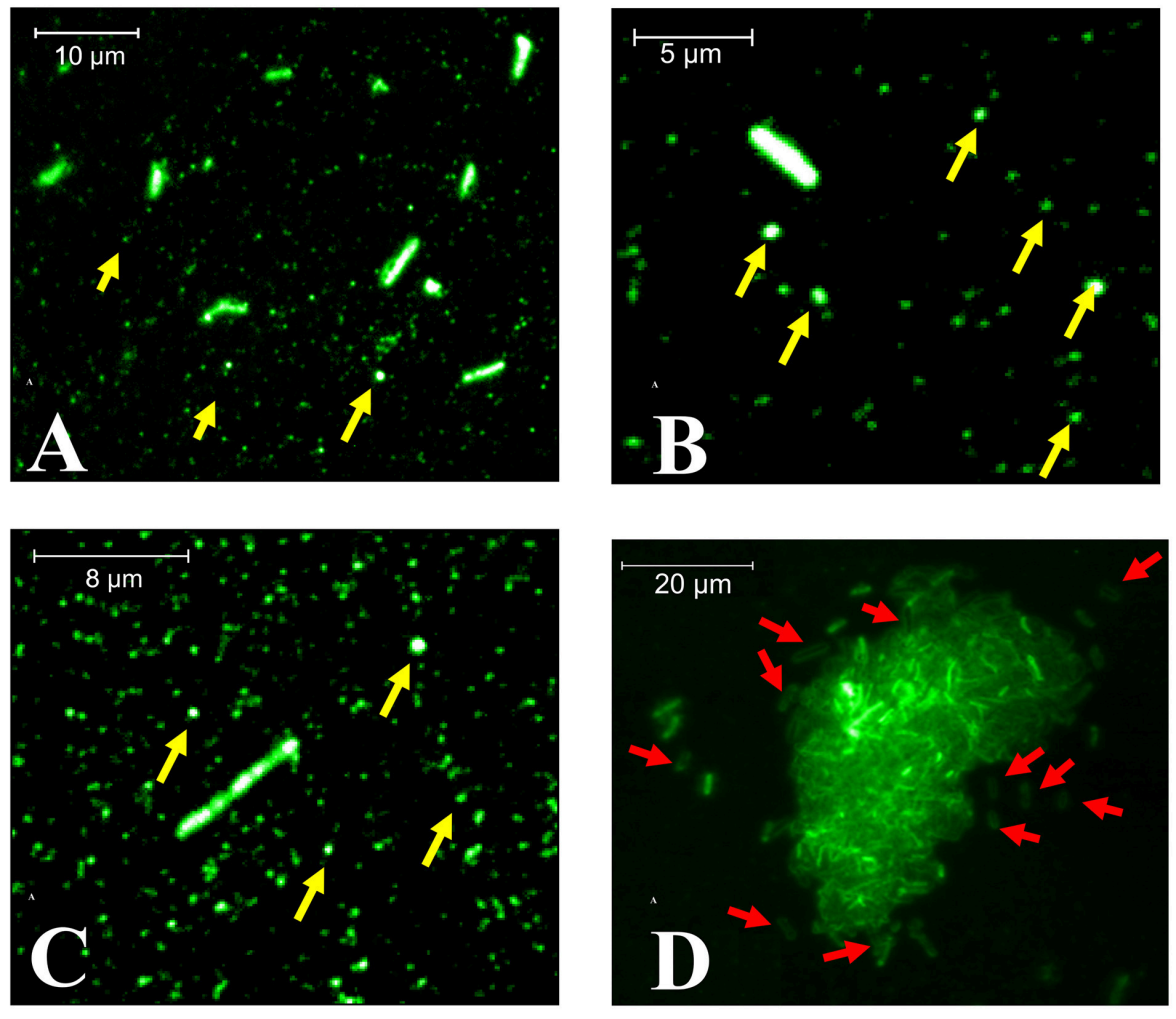
**Fluorescence microscopy of XfYgiT inside OMVs and cells of ***X. fastidiosa*** strain 9a5c**. The expression of XfYgiT within vesicles was evaluated using a polyclonal anti-XfYgiT antibody coupled to FITC. After growth in PW broth, the cells were transferred to borosilicate glasses and incubated for 4 more days to allow adhesion. The putative OMVs (yellow arrows) are indicated in **(A–C)**. In **(D)**, XfYgiT can be visualized in the periplasm of some cells (red arrows). All images were captured using a 100X oil-immersion objective (CFI APO TIRF, NA. 1.45, Nikon, USA).

After bacterial adhesion and sample preparation with anti-YgiT coupled to FITC, we could detect XfYgiT in the cells and in small points that we suggest were OMVs (Figure 6). Moreover, we could detect XfYgiT in small circles surrounding the cells, which we considered to be OMVs (Supplemental Figure 1). In some cells, XfYgiT could only be observed in the periplasm. After bacterial adhesion and sample preparation with anti-YgiT coupled to FITC, we could detect XfYgiT in the cells and in small points that we suggest were OMVs (Figure 6). Moreover, we could detect XfYgiT in small circles surrounding the cells, which we considered to be OMVs. In some cells, XfYgiT could only be observed in the periplasm. In the treatment with anti-XfPal in samples with bacteria, we detected its presence in the cells as well as in the OMVs (Supplemental Figure 2B). Labeling of XfPal also took place in samples with purified vesicles. This result suggests that staining takes place in the vesicles, and not parts of bacterial membrane due to the absence of bacterial cells (Supplemental Figure 2D). However, XfMqsR was detected in the cell as well as in the OMVs.

## Discussion

TA systems are widely distributed in bacteria and archaea (Park et al., 2013) and are considered to have been acquired by horizontal gene transfer because the genes are embedded in a W-V prophage region. However, it is unknown if this prophage retains the capacity to generate infectious phages in a lytic cycle (Lee et al., 2014).

Bacterial pathogenicity may be directly proportional to the number of TA systems in the genome compared with the genomes of species that are not involved in epidemics (Georgiades and Raoult, 2011). Several studies have demonstrated the participation of the TA system in biofilm formation (Fasani and Savageau, 2013; Van Acker et al., 2014; Wen et al., 2014). As demonstrated by Merfa et al. (2016), *X. fastidiosa* strain 11399 overexpressing XfMqsR is able to produce a large number of persister cells that can survive under copper stress, positively regulate biofilm formation, and repress cell movement.

Our work provides a detailed characterization of a TA system from the *X. fastidiosa* subsp *pauca* strain 9a5c.

The *orfs* identified in this study were previously classified as a hypothetical protein (XfMqsR) and an HTH-type transcriptional regulator (XfYgiT) in the *X. fastidiosa* database. Our results demonstrated that XfMqsR and XfYgiT have 99% similarity to their homologs in the closest related strain, Temecula, which infects grapevine, as demonstrated by Lee et al. (2014). Additionally, a comparison of XfMqsR and XfYgiT with MqsR and MqsA, their respective homologs in *E. coli*, revealed their predicted structures with 100% reliability. The formation of the TA complex was thermodynamically favorable, and the complex displayed a low dissociation constant. As demonstrated in a previous report (Lee et al., 2014), the use of a p-Duet vector, in which MqsR and YgiT from *X. fastidiosa* strain Temecula were induced and concomitantly purified using affinity chromatography, showed that these proteins were able to interact. However, the expression of the XfMqsR and XfYgiT in different vectors allowed us to establish the dissociation coefficient via isothermal titration calorimetry, to obtain the *Tm* values of the isolated proteins and of the complex, as well as and to identify the interaction stoichiometry.

XfYgiT was able to hinder the RNA activity of its cognate protein, and both proteins were differentially expressed at high levels during the biofilm phase. Additionally, XfYgiT was detected inside OMVs, as confirmed by Western blotting and fluorescence microscopy analyses; to some extent, this localization may be related to other likely unknown functions related to XfYgiT because this protein lacks a signal peptide. Further, studies should be conducted to elucidate the mechanism by which XfYgiT is translocated to the cell membrane to reach the periplasmic space.

The amino acid residues involved in RNAse activity were 100% conserved between XfMqsR and MqsR, its *E. coli* homolog. *E coli* cells containing the MqsR protein with mutations in the residues Lys^56^, Gln^68^, Tyr^81^, and Lys^96^ were less lethal due to a decrease in RNAse activity (Brown et al., 2009); these residues were conserved in *X. fastidiosa* (Figure 1). The activity of this protein was primarily localized to the β-sheet regions and involved RNAse activity; however, the primary sequence did not contain any similarity to other RNAse domains. In *E. coli* as well as *X. fastidiosa* strain Temecula, MqsR specifically cleaves RNAs primarily at 5′- G_▴_CU and G_▴_CC sites. (Christensen-Dalsgaard et al., 2010; Wang et al., 2012; Wen et al., 2014).

Although XfYgiT was less conserved relative to its *E. coli* homolog compared to XfMqsR, it possessed conserved domains related to its interaction with its promoter and the toxin. The residues Asn^97^ and Arg^101^, which are conserved in the *E. coli* and *X. fastidiosa* strain 9a5c proteins (Figure 1B), are involved in promoter recognition. Additionally, the region containing the HTH domain shares 59% similarity with the corresponding region in *E. coli*. According to a previous report (Brown et al., 2011), the antitoxin recognizes a motif sequence [TAACCT(N_3_)AGGTTA]. The promoter of the XfYgiT and XfMqrR operon displays a very similar sequence [TAACCT(N3)AAGTTA] located between positions −24 and −9 that may serve as the XfYgiT anchoring site (data not shown). The conserved residues Asn^97^ and Arg^101^ are responsible for the recognition of the following eight nucleotides: 5′ TAAC3′ in the forward strand and 5 “AGGT 3” in the reverse strand (Brown et al., 2011). Indeed, XfYgiT was able to interact specifically with its own promoter at 1:2 molar ratios and as described above (data not shown).

A hydrodynamic characterization of the heterologous purified proteins revealed that XfYgiT behaves as a dimer (31.6 kDa) and XfMqsR behaves as a monomer (16.9 kDa) *in solution*. This analysis was performed using SEC. The AUC suggested molecular weights of 32.5 and 10.1 for XfYgiT and XfMqsR, respectively. In *E. coli*, the TA interaction was found to occur at a 2:2 ratio. The complex consists of an antitoxin dimer and two toxin monomers, which is also known as a dimer of dimers (Wen et al., 2014). In the present study, an equimolar ratio was observed for the complex using SEC (Figure 2A). The AUC analysis confirmed the presence of XfYgiT in the dimeric form, with a molecular mass of 32.8 ± 0.2 kDa. XfMqsR was present in the monomeric form, with a molecular mass of 10.1 ± 0.35 kDa. The differences in the molecular mass observed during SEC analysis were due to the intrinsic traits of the technique used. SEC is more reliable for very globular proteins (den Engelsman et al., 2011). Thus, the AUC data offer a more specific result regarding the molecular mass of the proteins and their Stokes radiuses.

As seen in *E. coli* (Brown et al., 2009), the MqsA-MqsR complex occurs at a 2:2 molar ratio. In this complex, a dimer of MqsA is bound by two monomers of MqsR. An *in silico* analysis predicted an expected molecular mass of 58.12 kDa. The result obtained by AUC confirmed this estimate; the molecular mass observed by AUC was 58.3 ± 1.4 kDa, confirming an equimolar ratio.

The interaction between these proteins is thermodynamically favorable. The proteins form a very stable complex, as demonstrated by thermal circular dichroism and ITC. A thermostability analysis confirmed that additional heat was necessary to unfold the complex, exceeding the heat required to denature the isolated proteins. Similar results have been reported for the *E. coli* TA system (Brown et al., 2013). The interactions between the homologs from *X. fastidiosa* strain Temecula were found to be very stable and spontaneous, as previously described (Lee et al., 2014).

Based on the recent discovery that plant colonization by *X. fastidiosa* subsp *fastidiosa* strain Temecula is likely modulated by OMVs (Ionescu et al., 2014), we analyzed the secretome of strain 9a5c. This approach led us to identify the secreted proteins via MS/MS. Among the encountered proteins, we discovered four peptides that aligned with XfYgiT. In an *in silico* analysis using SignalP 4.1 software, we discovered that XfYgiT did not possess a leader sequence or a transmembrane domain, suggesting that the protein is located in the intracellular compartment.

However, the presence of XfYgiT in the extracellular media led us to hypothesize that it is secreted by vesicles; this hypothesis was confirmed by Western blotting of the fractions obtained by centrifugation to isolate the OMVs and by fluorescence microscopy. This is not the first study of the association between a predicted intracellular protein and OMV formation. For example, in *X. fastidiosa* subsp *fastidiosa* strain Temecula, a proteomic approach to the secretome identified 17 proteins without signal peptides (Nascimento et al., 2016). Other studies have identified proteins related to glycolysis and transcription factors inside OMVs (Altindis et al., 2014; Chen et al., 2014; Choi et al., 2014). The pathways used by *X. fastidiosa* subsp *pauca* strain 9a5c to secrete wild-type XfYgiT protein remain unknown, necessitating further studies to unravel the underlying mechanism.

OMVs are 20 to 300 nm in diameter (Ellis and Kuehn, 2010; Schertzer and Whiteley, 2013; Ionescu et al., 2014; Kuipers et al., 2015), and their secretion is related to the spread of *X. fastidiosa* throughout the plant xylem (Ionescu et al., 2014). OMVs have been reported to carry virulence factors and toxins (Ellis and Kuehn, 2010; Altindis et al., 2014); however, their role in pathogenicity is not completely understood (Kuehn and Kesty, 2005; Schertzer and Whiteley, 2013). OMVs are involved in the modulation of immune responses, the secretion of antibacterial compounds, and the facilitation of bacterial movement (Ionescu et al., 2014); they are also involved in protecting the cell from the surrounding environment by preventing other cells from colonizing the same site (Altindis et al., 2014; Ionescu et al., 2014; Kuipers et al., 2015).

The molecular mechanisms underlying vesicle formation and the mechanisms by which proteins become enveloped during vesicle formation remain unclear. Many hypotheses have been proposed concerning the formation of OMVs and their function (i.e., modulation of the microbial environment to kill competing species, signaling likely sites of nodulation for biofilm formation and modulating the immune response system in host cells; Choi et al., 2014; Schwechheimer et al., 2014; Pérez-Cruz et al., 2015; Turner et al., 2015). This study is the first to report the presence of XfYgiT inside OMVs (Supplemental Figures 2A,C). It is possible visualize the presence of XfYgiT in the periplasm in some cells; however, the pathway used for its transport is unclear (Figure 6). The presence of XfYgiT in the periplasm may be the first step of in their inclusion in OMVs during OMV formation.

The control chosen in this study, partially worked during our experiments. Anti-XfPal coupled to Atto 594 was present in the cells as well as in the OMVs (Supplemental Figure 2B). On the other hand, samples treated with anti-XfMqsR coupled to Atto 488 also marked cell and OMVs, demonstrating not to be suitable for negative control. Our hypothesis is that the lysozyme treatment disrupted the cell wall, making the toxin able to interact with antitoxin in the OMVs. To avoid this problem, we have tried to label the samples without lysozyme treatment, but we did not obtain satisfactory results. The alternative negative control of XfPal labeling in samples with purified vesicles showed the labeling of the small circles. The absence of bacteria in this sample suggests that staining takes place in vesicles and not in parts of bacterial membrane (Supplemental Figure 2D).

The microscopy investigation indicates that XfYgiT is secreted along with OMVs, since the fraction after 100,000 g centrifugation is formed almost exclusively by nanometric-size structures we assume to be the OMVs. In the SEM images, larger OMVs are observed probably due to the metallization of the sample, which can cover the smaller vesicles (Supplemental Figure 3). However, spherical structures with 10–35 nm in size are observed in the AFM images (Supplemental Figure 4). In the SEM images, a few spherical structures surround and are still bound to the cell surface; we assume these structures to be OMVs, as suggested by similar results from Ionescu et al. (2014). From fluorescence microscopy, we assume that these small fluorescent regions are the OMVs surrounding the *X. fastidiosa* cells. The microscopy results further support our hypothesis, because the centrifugation procedure provided homogeneous samples comprising essentially spherical structures we suggest to be OMVs.

Although further studies are necessary, we hypothesize that the release of XfYgiT into the extracellular media results in an excess accumulation of intracellular XfMqsR, which would produce favorable conditions for biofilm formation and the generation of persister cells, thus inducing pathogenicity. Further, studies are also needed to elucidate the mechanism used by *X. fastidiosa* strain 9a5c to secrete XfYgiT.

*X. fastidiosa* is an important phytopathogen, and the elucidation of its mechanisms of infection is crucial for the control of disease caused by this bacterium (Mansfield et al., 2012). In this work, we functionally characterized a toxin and an antitoxin belonging to a TA system from the *X*. *fastidiosa* subsp *pauca* strain 9a5c based on heterologous expression and analyzed their expression using Western blotting and hydrodynamic studies. Our results contribute to an understanding of proteins that participate in biofilm formation and perform other functions. XfYgiT was present inside OMVs, which indicates that it is involved in other functions that remain to be determined. Thus, further studies are needed to determine the influence of this TA system on biofilm formation and bacterial pathogenicity.

## Author contributions

ASS, JM, CS, MT, AAS, and APS contributed to the conception and design of the work; ASS, JM, CS, and MT performed the data acquisition; ASS, JM, CS, LB, AC, MH, MF, and APS analyzed and interpreted the data; DM and MC designed experiments, performed the microscopy and analyzed the data and, ASS, JM, CS, MT, AAS, MC, and APS drafted the article or revised it critically for important intellectual content. All authors read and approved the final manuscript.

### Conflict of interest statement

The authors declare that the research was conducted in the absence of any commercial or financial relationships that could be construed as a potential conflict of interest.

## Supplementary material

Supplementary Figure 1 **Fluorescence microscopy images of ***X. fastidiosa*** samples on glass substrate: Autofluorescence images of bacteria and vesicles (A)** and vesicles only **(C)**. Fluorescence image of samples labeled with polyclonal antibody against XfYgiT in outer membrane vesicles and cells **(B)** and in vesicles only **(D)**. The maximum intensity in the autofluorescence (3700 counts) is significantly lower than the minimum intensity in the labeled samples (6500 counts), demonstrating a much higher fluorescence efficiency after antibody binding. The images were acquired using the same parameters so that a quantitative intensity analysis could be carried out.

Supplementary Figure 2 **Labeling in samples with bacteria using anti-XfYgiT coupled to FITC (A)**, anti-PAL coupled to Atto 594 **(B)**. Labeling in samples with purified vesicles using anti-XfYgiT coupled to FITC **(C)**, anti-PAL coupled to Atto 594 **(D)**. These results suggest that the small circles are OMVs and not parts of bacterial membrane.

Supplementary Figure 3 **SEM images showing (A)** *X.fastidiosa* cells in a metal-coated sample grown on glass; **(B,C)** spherical structures attached to the cell surfaces (arrows); **(D,E)** spherical structures (arrows) in the sample in which centrifugation was previously used to eliminate the bacterial cells.

Supplementary Figure 4 **AFM images obtained for the sample in which centrifugation was used to eliminate the bacterial cells. (A)** topography and **(B)** phase images in a large area view. The change in phase signal at the spherical structures with sizes ~10nm suggests these structures are composed of materials with elastic features different from that of the background. **(C–E)** topography images of larger spherical structures (sizes ~20–30nm) observed for the same sample type.
