# Supplementary figures and images for: The Antitoxin Protein of a Toxin-Antitoxin System from Xylella fastidiosa Is Secreted via Outer Membrane Vesicles
Source: Front Microbiol. 2016 Dec 20;7:2030. doi: 10.3389/fmicb.2016.02030 (PMC5167779; doi:10.3389/fmicb.2016.02030)

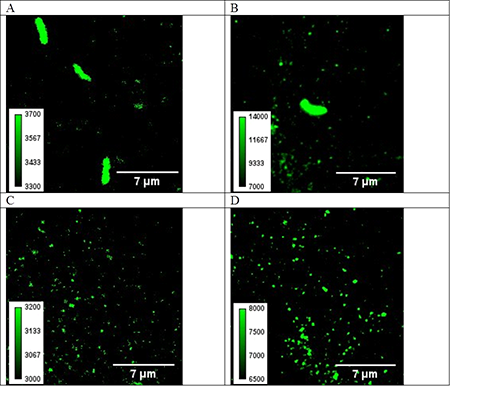

Supplement: Supplementary Figure 1 — Fluorescence microscopy images of X. fastidiosa samples on glass substrate: Autofluorescence images of bacteria and vesicles (A) and vesicles only (C). Fluorescence image of samples labeled with polyclonal antibody against XfYgiT in outer membrane vesicles and cells (B) and in vesicles only (D). The maximum intensity in the autofluorescence (3700 counts) is significantly lower than the minimum intensity in the labeled samples (6500 counts), demonstrating a much higher fluorescence efficiency after antibody binding. The images were acquired using the same parameters so that a quantitative intensity analysis could be carried out. [file Image1.TIF]

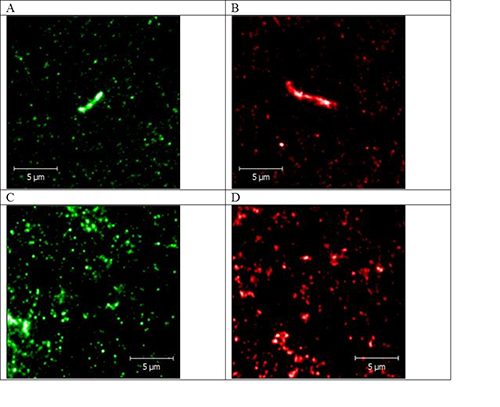

Supplement: Supplementary Figure 2 — Labeling in samples with bacteria using anti-XfYgiT coupled to FITC (A), anti-PAL coupled to Atto 594 (B). Labeling in samples with purified vesicles using anti-XfYgiT coupled to FITC (C), anti-PAL coupled to Atto 594 (D). These results suggest that the small circles are OMVs and not parts of bacterial membrane. [file Image2.TIF]

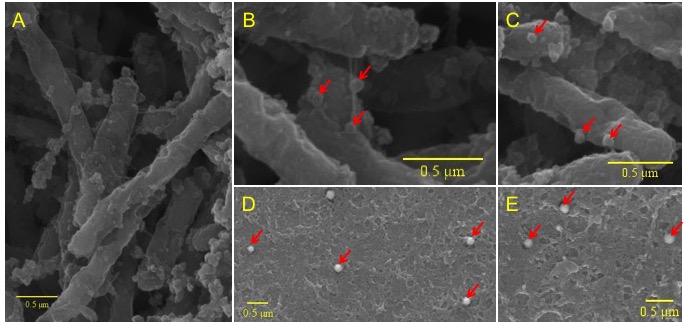

Supplement: Supplementary Figure 3 — SEM images showing (A) X.fastidiosa cells in a metal-coated sample grown on glass; (B,C) spherical structures attached to the cell surfaces (arrows); (D,E) spherical structures (arrows) in the sample in which centrifugation was previously used to eliminate the bacterial cells. [file Image3.JPEG]

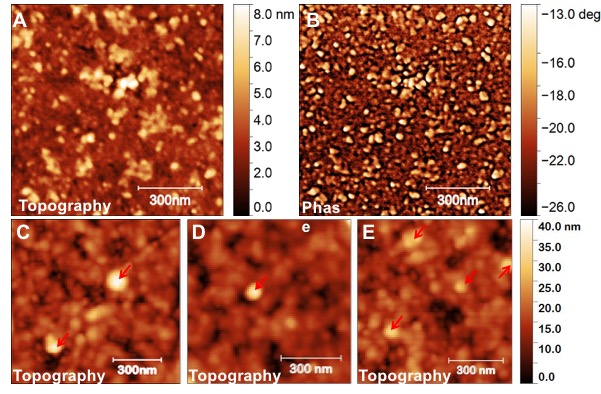

Supplement: Supplementary Figure 4 — AFM images obtained for the sample in which centrifugation was used to eliminate the bacterial cells. (A) topography and (B) phase images in a large area view. The change in phase signal at the spherical structures with sizes ~10nm suggests these structures are composed of materials with elastic features different from that of the background. (C–E) topography images of larger spherical structures (sizes ~20–30nm) observed for the same sample type. [file Image4.JPEG]
